# Operational properties of fluctuation X-ray scattering data

**DOI:** 10.1107/S2052252515002535

**Published:** 2015-03-20

**Authors:** Erik Malmerberg, Cheryl A. Kerfeld, Petrus H. Zwart

**Affiliations:** a Physical Biosciences Division, Lawrence Berkeley National Laboratory, Berkeley, California, USA; b Department of Plant and Microbial Biology, UC Berkeley, Berkeley, California, USA; c DOE Plant Research Laboratory, Michigan State University, East Lansing, Michigan, USA

**Keywords:** fluctuation X-ray scattering, XFELS, biological molecules, nanoparticles, mesoscopic materials

## Abstract

X-ray scattering images collected on timescales shorter than rotation diffusion times using a (partially) coherent beam result in a significant increase in information content in the scattered data. These measurements, named fluctuation X-ray scattering (FXS), are typically performed on an X-ray free-electron laser (XFEL) and can provide fundamental insights into the structure of biological molecules, engineered nanoparticles or energy-related mesoscopic materials beyond what can be obtained with standard X-ray scattering techniques. In order to understand, use and validate experimental FXS data, the availability of basic data characteristics and operational properties is essential, but has been absent up to this point. In this communication, an intuitive view of the nature of FXS data and their properties is provided, the effect of FXS data on the derived structural models is highlighted, and generalizations of the Guinier and Porod laws that can ultimately be used to plan experiments and assess the quality of experimental data are presented.

## Abstract 2

X-ray scattering images collected on timescales shorter than rotation diffusion times using a (partially) coherent beam result in a significant increase in information content in the scattered data. In this communication, an intuitive view of the nature of fluctuation scattering data and their properties is provided, the effect of such data on the derived structural models is highlighted, and generalizations of the Guinier and Porod laws that can ultimately be used to plan experiments and assess the quality of experimental data are presented.

## 1. Introduction   

In biology, materials science and the energy sciences, structural information provides important insights into the understanding of matter. The link between a structure and its properties can suggest new avenues for designed improvements of materials, nanoparticles and proteins. For samples without long-range order, such as solutions of biological macromolecules, disordered organic polymers or magnetic domains, as well as (partially) ordered materials, such as self-assembled block copolymers, liquid crystals or assemblies of nanoparticles, structural information can be obtained efficiently using traditional small- and wide-angle X-ray scattering (SAXS/WAXS) techniques (Gann *et al.*, 2012 ▶; Dyer *et al.*, 2014 ▶). Samples lacking long-range order typically display angular isotropic X-ray scattering patterns, where the mean intensity as a function of scattering angle is directly related to the average shape and local organization of the material investigated (Feigin *et al.*, 1987 ▶; Glatter & Kratky, 1982 ▶).

The isotropic nature of these SAXS/WAXS diffraction patterns is a result of orientational averaging of the scattering species, due to the fact that the timescale of X-ray exposure exceeds that of rotational diffusion. The advent of coherent X-ray sources (Emma *et al.*, 2010 ▶; Ishikawa *et al.*, 2012 ▶; Vartanyants *et al.*, 2007 ▶; Feldhaus *et al.*, 2013 ▶; Borland, 2013 ▶) such as free-electron lasers (FELs) and ultra-bright synchrotron light sources allows one to reduce the exposure timescale below that of rotational diffusion such that the non-isotropic intensity fluctuations (or speckle) in the scattering pattern can be resolved.

The first experimental demonstration of this technique, termed by the inventor (Kam, 1977 ▶) as fluctuation X-ray scattering (FXS), was provided by Kam *et al.* (1981 ▶) on frozen tobacco mosaic virus in the early days of synchrotron-based small-angle scattering. Subsequently, fluctuation scattering has been used to detect hidden symmetries in colloids (Wochner *et al.*, 2009 ▶) and magnetic domains (Su *et al.*, 2011 ▶), for the structure determination of two-dimensional particles (Pedrini *et al.*, 2013 ▶; Chen *et al.*, 2012 ▶; Saldin, Poon *et al.*, 2010 ▶), and for the characterization of liquid crystals (Kurta *et al.*, 2013 ▶) and glasses (Cowley, 2001 ▶). XFEL-based fluctuation (X-ray) scattering data and structure determination have been demonstrated from single and multiple inorganic nanoparticles (Liu *et al.*, 2013 ▶; Mendez *et al.*, 2014 ▶) and single polystyrene dumb-bells (Starodub *et al.*, 2012 ▶).

Information is extracted from the experimental speckle patterns by computing in-frame angular intensity correlations (Kam, 1977 ▶; Saldin, Poon *et al.*, 2010 ▶; Saldin, Poon, Bogan *et al.*, 2011 ▶; Saldin *et al.*, 2009 ▶). These angular intensity correlation curves, the FXS data, can be used for structure determination, either *via* reciprocal-space techniques (Poon *et al.*, 2013 ▶; Saldin, Poon, Schwander *et al.*, 2011 ▶; Saldin, Shneerson *et al.*, 2010 ▶) or *via* real-space methods (Chen *et al.*, 2012 ▶; Liu *et al.*, 2013 ▶). In earlier studies, FXS has been presented as a method for overcoming experimental and theoretical hurdles in single-particle imaging (Kam, 1977 ▶; Saldin *et al.*, 2009 ▶). In contrast with this viewpoint, we demonstrate here that FXS is a natural extension of SAXS/WAXS. Despite the increased attention paid to fluctuation scattering due to newly constructed and future light sources, there is a significant lack of understanding of the basic properties of such data. The absence of a basic grasp of the general nature and characteristics of the data makes assessment, validation and proper use of the experimental data a challenge.

This communication will provide an in-depth view of the nature of fluctuation X-ray scattering data, resulting in the derivation of Guinier and Porod relations and other operational properties. We furthermore present the effect of the progressive inclusion of FXS data when reconstructing three-dimensional models, demonstrating the superior quality of models that can be obtained from limited FXS data. The benefits of FXS data apply not only to low-resolution shape or structure determination, but extend to model-based structural refinements as well, allowing one to determine structural changes due to ligand binding or other externally induced perturbations.

## 2. Results and discussion   

### 2.1. FXS extends traditional small- and wide-angle X-ray scattering   

The diffraction pattern of an ensemble of molecules frozen in space and time will contain the signature of many particles, combining effects from the shape and internal structure of the particles, the so-called form factor, and their mutual arrangement in space, the structure factor. In the case of an ideal dilute solution, one can show that the mean angular intensity correlation function, *C*_2_(*q*, Δϕ), averaged over a large number of independent multiple-particle shots, is equivalent to that obtained from single-particle data (Kam, 1977 ▶; Saldin *et al.*, 2009 ▶), assuming no interparticle inter­actions (Kam, 1977 ▶; Saldin *et al.*, 2009 ▶; Kirian *et al.*, 2011 ▶; Altarelli *et al.*, 2010 ▶) and the presence of a flat X-ray wavefront during the scattering process (Lehmkühler *et al.*, 2014 ▶; Schroer *et al.*, 2014 ▶). The potential effects of the coherence properties of the X-ray beam on the resulting angular correlations will be discussed elsewhere.

### 

The angular correlation function can be obtained from the experimental data by averaging a large number of in-frame intensity correlation functions 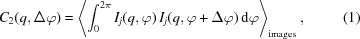
where *I*_*j*_(*q*, ϕ) denotes the intensity as recorded on the *j*-th diffraction pattern at polar coordinate (*q*, ϕ) [*q* = (4π/λ)sinθ, where θ is half the scattering angle and λ is the wavelength of the incident radiation]. Note that additional cross-resolution and *n*-point correlations can be derived as well (Kam, 1977 ▶) but are not considered at this point.

### 

The function *C*_2_(*q*, Δϕ) can be further decomposed into orthogonal components 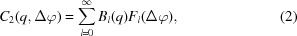
where *B*_*l*_(*q*) are resolution-dependent weights and *F*_*l*_(Δϕ) is given by 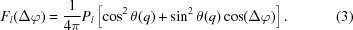 Here, *P*_*l*_(·) is a Legendre polynomial and 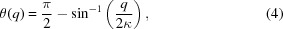
where κ is equal to the wavenumber 2π/λ, with λ the wavelength of the incident radiation. Note that, due to Friedel’s law, *B*_*l*_(*q*) terms for odd *l* are equal to 0 (Kam, 1977 ▶).

### 

The set of resolution-dependent expansion coefficients *B*_*l*_(*q*), as obtained from the experimental data, is related to the three-dimensional structure ρ(**x**) (Kam, 1977 ▶; Saldin *et al.*, 2009 ▶). Although the derivation relating the three-dimensional structure to the expansion coefficients *B*_*l*_(*q*) is relatively straightforward, it does not provide an intuitive insight into the nature of the data.

### 

**Figure 1:**
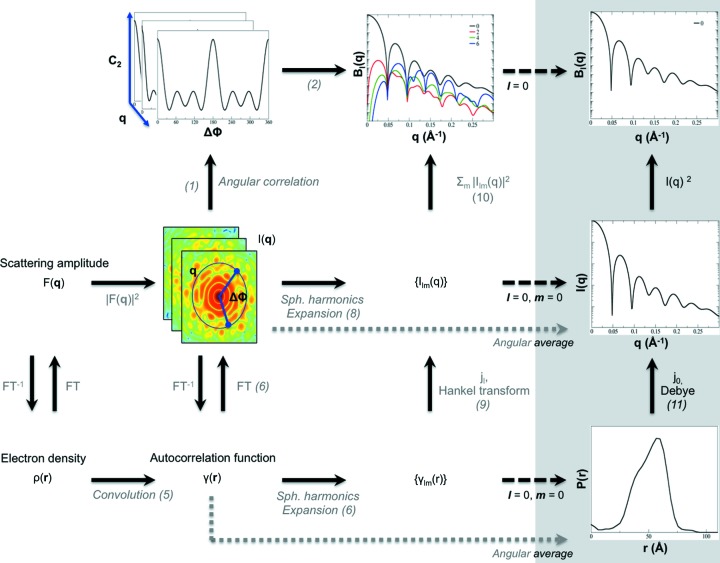
The ‘magic square’ of scattering is expanded to show the relation between the real-space electron density ρ(**r**), the associated autocorrelation function γ(**r**) and its Fourier transforms, *F*(**q**) and *I*(**q**), respectively. When expressing γ(**r**) and *I*(**q**) in a spherical coordinate system, Hankel transforms relate the associated expansion coefficients. Orientation-averaged quantities in the grey column, such as SAXS data and the radial distance distribution, can be obtained by selecting curves for which *l* = 0. The numbers in parentheses relate key operations to the corresponding equations given in the text.

Traditionally, fluctuation scattering data are presented starting from the Fourier transform of the real-space structure of the sample (Kam, 1977 ▶). Additional insights are obtained when following the route typically used to derive standard relations in small-angle X-ray and neutron scattering. A graphical depiction of fluctuation scattering and how it is related to standard SAXS is shown in Fig. 1 ▶, in which the mathematical relations outlined below are referenced. Starting from the real-space structure ρ(**x**), the Patterson function γ(**u**) can be obtained *via* a self-convolution 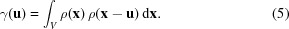
By switching to a spherical coordinate system and expressing the Patterson function as a spherical harmonics series, we obtain 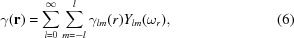
where γ_*lm*_(*r*) are the expansion coefficient curves of the real-space autocorrelation function and *Y*_*lm*_(·) is a spherical harmonic function. Given that the scattered intensity is proportional to the Fourier transform of the real-space autocorrelation function, one has 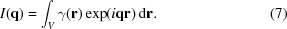
Expressing this intensity function as a spherical harmonics series 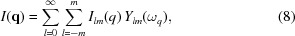
one obtains (Baddour, 2010 ▶) 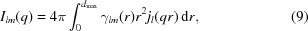
where *j*_*l*_(·) is a spherical Bessel function of order *l*. These intensity function expansion coefficients are related to the fluctuation scattering curves *B*_*l*_(*q*) *via* (Kam, 1977 ▶; Saldin *et al.*, 2009 ▶) 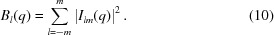
From the above equations and Fig. 1 ▶, it is clear that fluctuation scattering is a natural extension of small-angle X-ray scattering.

### 

In the analysis of traditional SAXS data, the system is assumed to be statistically isotropic, resulting in the assumption that coefficients *I*_*lm*_(*q*) for *l* > 0 are not experimentally accessible. The term *I*_00_(*q*) is of course equal to SAXS data, as it models the mean intensity as a function of momentum transfer *q*. Upon further inspection of equation (9) for *l* = 0, we obtain the Debye equation 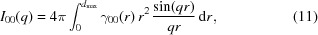
where γ_00_(*r*)*r* ^2^ can be recognized as the pair distance distribution function *P*(*r*) (Feigin *et al.*, 1987 ▶; Glatter & Kratky, 1982 ▶).

### 

Whereas SAXS data only provide experimental information about the zero-order polar Fourier transform of the real-space autocorrelation of the real-space object [equation (11)], fluctuation scattering extends the data into higher-order descriptors of the sample. Given that both SAXS and fluctuation scattering data can be described as *l*-th order spherical Hankel transforms of radial expansion coefficients, it should come as no surprise that certain operational properties from SAXS data can easily be expanded into the fluctuation scattering framework.

## 3. Guinier and Porod laws for FXS data   

**Figure 2:**
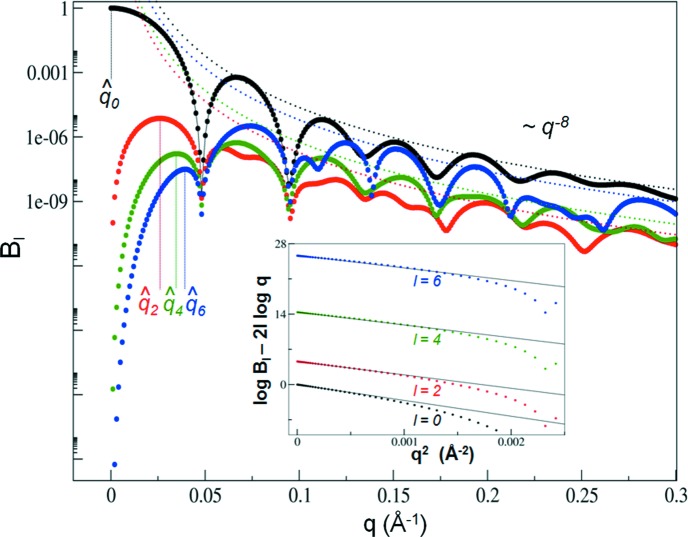
Model *B*_*l*_(*q*) coefficients from STMV for *l* = 0 (black), 2 (red), 4 (green) and 6 (blue). The inset depicts generalized Guinier plots with linear fits. The location of the first maximum 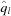 in *B*_*l*_(*q*), as obtained from the Guinier analysis, is indicated. The Porod behaviour of the data, characterized by an asymptotic fall-off proportional to *q* ^−8^, is shown by dotted lines.

As is the case for SAXS data, the low-resolution behaviour of fluctuation scattering data can provide insights into the structural parameters in a model-free fashion and can be used to check the general quality of the data. Using an infinite series expression for spherical Bessel functions (Bowman, 1958 ▶) in equation (9) and truncating the series to the second term, as done when deriving the standard Guinier relation, one quickly obtains 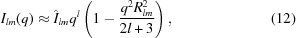
where 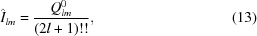


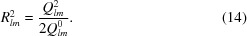
The quantities 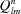 are the *n*-th order multipole moments of the autocorrelation function 
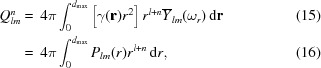
with *P*_*lm*_(*r*) = γ_*lm*_(*r*)*r* ^2^. 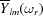 denotes complex conjugation of the spherical harmonic *Y*_*lm*_(ω_*r*_). Note that, in general, *I*_*lm*_(*q*), 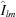 and 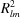 are complex quantities unless *l* = 0. Equation (12) can be substituted into equation (10), ultimately resulting in 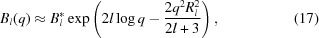
where 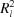 is equal to the mean real part of 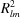 (−*l* ≤ *m* ≤ *l*) and 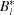 is related to the average absolute value of 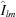 for a fixed value of *l*. Linearizing this expression yields a generalized Guinier plot 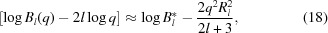
where the slope and intercept provide information on the sample-dependent properties 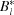 and 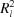. From this general formulation of the Guinier equation, it now becomes evident that 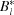 and 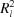 represent the average amplitudes of the zero- and second-order multipole moments, 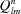. For 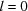, *i.e.* a monopole, this is synonymous with the square of the total scattering length, *I*(0)^2^, and the squared radius of gyration, 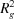, of the particle. For *l* > 0, these two quantities likewise describe the higher-order moments (quadrupoles, hexadecapoles *etc.*) of the particle shape. The relative magnitudes of these invariants for different values of *l* are influenced by the symmetry of the particle, leading to systematic absences of *B*_*l*_(*q*) (Saldin, Poon, Schwander *et al.*, 2011 ▶). A generalized Guinier plot from synthetic data is shown in Fig. 2 ▶, using satellite tobacco mosaic virus (STMV) as an example.

The generalized Guinier equation also allows one to estimate the location of the first local maximum 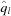 in *B*_*l*_(*q*), such that 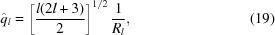
where the height, 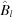, can be shown to be equal to 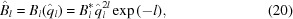
Although 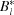 and 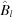 are related, the latter quantity is on a similar numerical scale to the total scattering length, making the use of this quantity more intuitive. 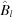 can be made scale-invariant by normalizing the data such that *B*_0_(*q*) = 1, which is assumed in the following paragraphs.

**Figure 3:**
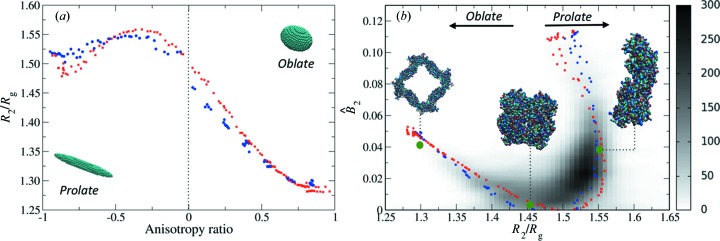
(*a*) Example shape descriptors for *l* = 2. The ratio *R*_2_/*R*_g_ is plotted against the anisotropic ratio for ellipsoids (black dots) or cylinders (red squares), allowing the identification of prolate or oblate features. (*b*) Including the use of 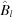 as a shape classifier, normalized against 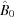, provides further discriminative power between shapes. Small values of 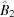 represent approximately spherical particles, while large values represent either prolate or oblate particles. The density in part (*b*) represents the empirical distribution of 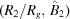 pairs, as obtained from known PDB structures (see Appendix A[App appa] for details).

The values 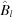 and *R*_*l*_ can be used as model-free shape classifiers beyond what is provided by the radius of gyration (*R*_g_ = *R*_0_) as obtained from a standard Guinier analysis. This is exemplified for *l* = 2 in Fig. 3 ▶, where the 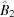 values and *R*_2_/*R* *g* ratio have been computed for a number of different sized cylinders, ellipsoids and a representative set of 6709 protein assemblies from the Protein Data Bank (Berman *et al.*, 2000 ▶) (see Appendix A[App appa] for details). From the cylinder and ellipsoid data, it is evident that the combination of *R*_2_/*R*_g_ and 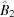 provides a combination of unique shape classifiers that allows one to distinguish prolate from oblate structural features. The value of *R*_2_/*R*_g_ indicates whether a shape has prolate or oblate characteristics, while the value of 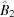 measures the extent or strength of the anisotropy, as large values of 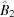 indicate significant deviations from sphericity. Higher-order moments can be used to expand this formalism further to provide a more fine-grained shape classification.

The above generalized Guinier analysis characterizes fluctuation scattering curves at low resolution. For SAXS/WAXS data, high-resolution data trends are described by Porod’s law: 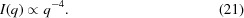
This trend holds for well defined three-dimensional particles. Following Porod’s derivation (Feigin *et al.*, 1987 ▶; Glatter & Kratky, 1982 ▶), but using the *l*-th order spherical Hankel transform and with an asymptotic approximation for *j*_*l*_(·) for large *q* (Bowman, 1958 ▶), one readily obtains 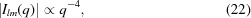
and one can thus show that, for large *q* values, Porod’s law extends to fluctuation scattering data 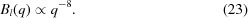
An illustration of this trend for STMV is depicted in Fig. 2 ▶.

**Figure 4:**
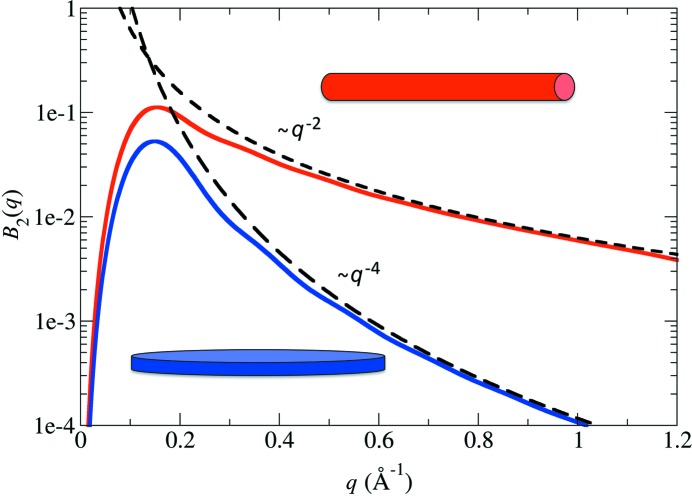
The Porod behaviour of FXS data for one-dimensional rods (red) and two-dimensional discs (blue) follows the same fall-off trends as seen in SAXS/WAXS data. Curves for *l* = 2 are shown; similar trends for higher-order curves exist.

The Porod behaviour of shapes such as discs [*B*_*l*_(*q*) ∝ *q* ^−4^] and rods (*B*_*l*_ ∝ *q* ^−2^) also displays the same characteristic fall-off (Feigin *et al.*, 1987 ▶; Glatter & Kratky, 1982 ▶) as expected for squared SAXS intensities (Fig. 4 ▶). A practical use of the predicted Porod behaviour is to use the expected fall-off as an inverse weight when fitting molecular or bead models to data, as is done in SAXS studies (Svergun, 1999 ▶).

This combination of Guinier and Porod analyses provides a set of model-independent tools to characterize and validate the quality of the experimental data, in the same way that Guinier and Porod analyses are used in biological small- and wide-angle scattering (Feigin *et al.*, 1987 ▶; Glatter & Kratky, 1982 ▶). The tools presented here provide straightforward guidelines for the evaluation of experimental FXS data or can be used to plan FXS experiments. An example of the use of the generalized Guinier analysis is the prediction that the first maximum in *B*_2_(*q*), 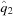, is expected to lie between 2.2/*R*_g_ and 1.6/*R*_g_ (see Appendix A[App appa]). If an *R*_g_ estimate is available from standard synchrotron SAXS studies, its value can be used in the experimental design of FEL-based experiments, to ensure that high-quality low-angle FXS data can be obtained.

## 4. Increased information content   

**Figure 5:**
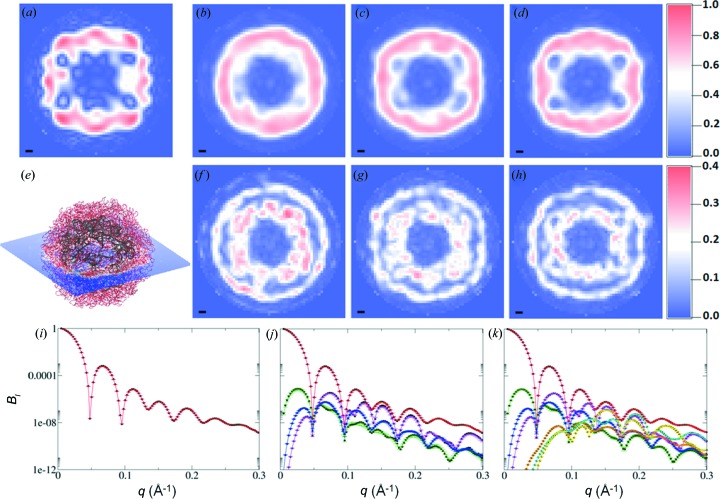
*Ab initio* unconstrained shape reconstructions from model STMV data. The reconstructions were performed by including *l* = 0 (*i.e.* SAXS data) [parts (*b*), (*f*) and (*i*)], *l* ≤ 6 [parts (*c*), (*g*) and (*j*)] or *l* ≤ 12 [parts (*d*), (*h*) and (*k*)] in the *B*_*l*_ refinement. The top row [parts (*a*)–(*d*)] depicts density slices through the centre of the virus (*e*). The reference density (*a*) shows significant detail in the core of the virus, which is largely absent when only SAXS data are used (*b*) but which is reproduced, with increasing quality, when terms up to *l* = 6 (*c*) and *l* = 12 (*d*) are considered. Another striking improvement is the distinctly non-spherical outer boundary of the particle when fluctuation scattering data are used. The second row [parts (*f*)–(*h*)] displays the associated standard deviations in the electron density as obtained from the ten independent aligned reconstructions. The black bar [parts (*a*)–(*d*) and (*f*)–(*h*) represents 10 Å. The bottom row [parts (*i*)–(*k*)] shows the agreement between the data (black circles) and the MOSA-refined (multi-objective simulated annealing; see Appendix A[App appa]) expansion coefficients [*B*_0_(*q*) red, *B*_2_(*q*) green, *B*_4_(*q*) blue, *B*_6_(*q*) magenta, *B*_8_(*q*) orange, *B*_10_(*q*) cyan and *B*_12_(*q*) yellow] for SAXS [part (*i*)] and for fluctuation scattering [parts (*j*)–(*k*)]. The error bars represent the standard deviation from the ten reconstructions.

The derivation of the basic properties of FXS data allows one to characterize and evaluate the quality of the experimental data, but fails to explain the reason why these types of experiment are beneficial. The principal advantage of FXS, as shown in Fig. 1 ▶, is the additional data made accessible in a fluctuation scattering experiment. This increase in experimental information, even in limited *q* and *l* ranges, allows the recovery of more structural detail compared with using *B*_0_(*q*), *i.e.* the SAXS data alone, in the same *q* range. This effect is illustrated in Fig. 5 ▶, in which average *ab initio* reconstructions obtained from both SAXS and FXS data are shown. The reconstructions are compared with the reference density from which the calculated data were obtained (see Appendix A[App appa] for details). The reconstructions and analyses here are limited to a relatively low order of *l*, since these curves are experimentally more easily accessible, and thus provide a conservative overview of the benefit of FXS data compared with standard SAXS data. As is clear from Fig. 5 ▶, the addition of limited higher-order scattering information already provides a spectacular increase in reproducible details in the proposed models. One of the reasons why we do not recover the target structure in an error-free fashion is that the optimization problem is still under-constrained (Elser, 2011 ▶). However, the main benefit is that FXS is able to reconstruct or derive structural details with greater confidence than can be accomplished from the SAXS data alone, ultimately leading to a better understanding of the structure-related properties.

**Figure 6:**
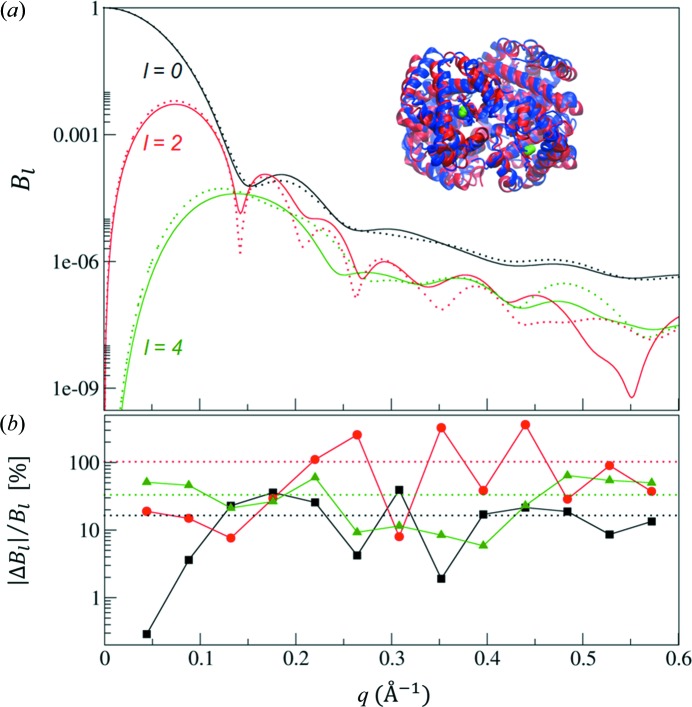
(*a*) FXS data calculated from the two haemoglobin crystal intermediates 1bbb (CO-haemoglobin; green and red cartoon and dotted lines) and 2hbb (deoxy-haemoglobin; blue cartoon and solid lines) in the Protein Data Bank. The average root mean-square difference between the two intermediates was approximately 2 Å and data were computed for *l* ≤ 4 (black, red and green curves). (*b*) The relative differences, |Δ*B*_*l*_(*q*)|/*B*_*l*_(*q*), between the two states at the Shannon sampling points (multiples of π/*d*_max_ = 0.044 Å^−1^) (black squares, red circles and green triangles), indicate the average increased sensitivity of *B*_*l*_(*q*) for *l* > 0, as illustrated by the dotted lines. This additional sensitivity, combined with the independent nature of the higher-order curves, ultimately results in a more precise determination of macromolecular structures in solution.

A similar view of the use of FXS data is obtained when we consider model-based refinement techniques for SAXS/WAXS data (Petoukhov & Svergun, 2005 ▶; Gorba & Tama, 2010 ▶). Given the stark differences in results obtained in *ab initio* modelling (Fig. 5 ▶), the further addition of geometric restraints from a known molecular model could resolve structural ambiguities to such a level that physiologically relevant conformational changes in macromolecules could be confidently deduced from FXS data. For example, when assuming that the structure of a resting state is known, an FXS experiment on the perturbed molecule can provide significantly more data than can be obtained from a SAXS experiment alone. This is illustrated in Fig. 6 ▶, in which model FXS data from carbon monoxide-bound haemoglobin are compared with its unligated intermediate. The relative difference in the data at the Shannon sampling points (Feigin *et al.*, 1987 ▶) is depicted as well, indicating that the sensitivity of *B*_*l*_(*q*) is enhanced for larger values of *l*. If high-order *l* data up to larger scattering angles are available, difference maps can be obtained as well (Pande *et al.*, 2014 ▶). It is worth noting that the extraction of a difference FXS signal will require optimal instrumental and sample conditions, as well as fine-tuned data-processing routines.

This increased information content of FXS data compared with SAXS can play an important role in determining the structural foundation of dynamic processes in biology. As shown earlier (Chen *et al.*, 2013 ▶), FXS from a mixture can be described as the component-weighted sum of curves from the individual species. By performing time-resolved FXS experiments, one can obtain *B*_*l*_(*q*) curves for intermediate short-lived structural species, akin to standard practices in the analysis of time-resolved WAXS data at synchrotrons (Cammarata *et al.*, 2008 ▶; Andersson *et al.*, 2009 ▶) or, as recently demonstrated, at an FEL (Arnlund *et al.*, 2014 ▶). Thus, the use of fluctuation scattering will ultimately lead to a more accurate depiction of the structural dynamics of macromolecules in solution.

## 5. Conclusions   

In conclusion, we have shown that fluctuation scattering is a natural extension of traditional small-angle X-ray scattering, and that a number of operational properties translate from SAXS/WAXS into fluctuation scattering. Given the increased detail that can be obtained from fluctuation scattering data and the ever-increasing availability of X-ray sources at which these experiments can be performed, we expect that these experiments will become routine in the future. The extended standard Guinier and Porod methods can be used to validate data and characterize samples rapidly in a model-free fashion.

## Appendix A Additional details

The cylinder and ellipsoid models used in Fig. 3 ▶ were obtained by generating voxelized representations of these shapes on a 41 × 41 × 41 voxel cubic grid with 40 Å edges. The set of shapes was obtained by varying their radii and lengths (cylinders) or their main and minor axes (ellipsoids) while keeping a constant volume. The anisotropy ratio as used in Fig. 3 ▶ is defined as

where λ_*z*_ is the moment of inertia, along the axis of revolution for ellipsoids or the cylindrical axis for cylinders. λ_*x*_ is the moment of inertia perpendicular to the *z* axis. The anisotropy ratio is 1 for a perfect one-dimensional rod and −1 for a two-dimensional disc. Fig. 3 ▶ indicates that *R*_2_/*R*_g_ is expected to lie between 1.25 and 1.65. Using equation (19), it follows that the first maximum in *B*_2_(*q*), , lies between 2.2/*R*_g_ and 1.6/*R*_g_. When determining *R*_*l*_ and  from FXS curves for *l* > 0, one can either use interpolation and peak picking and equation (19), or use the generalized Guinier transform, equation (18).

The empirical distribution , as shown in Fig. 3 ▶(*b*), was obtained from 6709 PDB files with low (<30%) sequence identity. The distribution shown contains 98% of the density. A small number of structures displayed *R*_2_/*R*_g_ ratios below 1.25 or above 1.65, with  typically close to zero.

All FXS data were computed from either the atomic co­ordinates (PDB models) or the electron density (real-space reconstructions) or voxelized representations (cylinders and ellipsoids) using the three-dimensional Zernike polynomial expansion method (Liu *et al.*, 2012 ▶). The maximum expansion order, *n*_max_, was determined such that *n*_max_ ≥ *q*_max_ *r*_max_, which resulted in *n*_max_ = 30 for STMV and *n*_max_ = 40 for haemoglobin. The *B*_*l*_(*q*) coefficients were evaluated to a maximum momentum transfer, *q*_max_, of 0.3 Å^−1^ (∼20 Å) for STMV (PDB code 1a34) and 0.6 Å^−1^ (∼10 Å) for haemoglobin (PDB codes 1bbb and 2hbb). The *ab initio* reconstructions were obtained without the use of symmetry or connectivity restraints *via* a multi-objective simulated annealing (MOSA) (Smith *et al.*, 2008 ▶) adaptation of our reverse Monte Carlo procedure (Liu *et al.*, 2013 ▶), which has been shown to be less prone to local minima than when using an aggregated χ^2^-based target function. The starting model for the reconstruction was a hollow sphere with a radius of 96 Å on a 61 × 61 × 61 voxel cubic grid.
